# Suspension microarray-based comparison of oropharyngeal swab and bronchoalveolar lavage fluid for pathogen identification in young children hospitalized with respiratory tract infection

**DOI:** 10.1186/s12879-020-4900-8

**Published:** 2020-02-22

**Authors:** Zhan-Ying Ma, Hua Deng, Li-Dong Hua, Wen Lei, Chang-Bin Zhang, Qi-Qiang Dai, Wei-Jing Tao, Liang Zhang

**Affiliations:** 1 Dongguan Maternal and Child Health Care Hospital, Dongguan, 523120 China; 2 Translational Medicine Center, Guangdong Women and Children Hospital, Guangzhou, 511400 China; 3 Prenatal Diagnosis Centre, Guangdong Women and Children Hospital, Guangzhou, 511400 China; 4 Guangzhou DaAn Clinical Laboratory Center, YunKang Group, Guangzhou, 51000 China

**Keywords:** Childhood respiratory illness, Oropharyngeal swab, Bronchoalveolar lavage fluid, Multipathogen detection, Suspension microarray

## Abstract

**Background:**

Respiratory tract infection (RTI) in young children is a leading cause of morbidity and hospitalization worldwide. There are few studies assessing the performance for bronchoalveolar lavage fluid (BALF) versus oropharyngeal swab (OPS) specimens in microbiological findings for children with RTI. The primary purpose of this study was to compare the detection rates of OPS and paired BALF in detecting key respiratory pathogens using suspension microarray.

**Methods:**

We collected paired OPS and BALF specimens from 76 hospitalized children with respiratory illness. The samples were tested simultaneously for 8 respiratory viruses and 5 bacteria by suspension microarray.

**Results:**

Of 76 paired specimens, 62 patients (81.6%) had at least one pathogen. BALF and OPS identified respiratory pathogen infections in 57 (75%) and 49 (64.5%) patients, respectively (*P* > 0.05). The etiology analysis revealed that viruses were responsible for 53.7% of the patients, whereas bacteria accounted for 32.9% and *Mycoplasma pneumoniae* for 13.4%. The leading 5 pathogens identified were respiratory syncytial virus, *Streptococcus pneumoniaee*, *Haemophilus influenzae*, *Mycoplasma pneumoniae* and adenovirus, and they accounted for 74.2% of etiological fraction. For detection of any pathogen, the overall detection rate of BALF (81%) was marginally higher than that (69%) of OPS (*p* = 0.046). The differences in the frequency distribution and sensitivity for most pathogens detected by two sampling methods were not statistically significant.

**Conclusions:**

In this study, BALF and OPS had similar microbiological yields. Our results indicated the clinical value of OPS testing in pediatric patients with respiratory illness.

## Background

Respiratory tract infection (RTI) in children is a leading cause of morbidity and hospitalization [1, 2]. Especially, severe pneumonia ranks the second most common cause of mortality in children younger than 5 years worldwide according to a recent report [3]. Viral and bacterial infections are the primary etiology of RTI. Accurate and rapid identification of the etiologic agents has an essential role in ensuring the appropriate and effective treatment for patients with respiratory illness, which could avoid unnecessary usage of antibiotics, reduce the overall costs and shorten the period of hospitalization [4].

Laboratory diagnosis of respiratory infections is performed traditionally using culture and immunological assays. Although microbial culture is regarded as the gold standard, it is time-consuming and labor-intensive. Specially, some fastidious pathogens are difficult to cultivate. Antigen/antibody detection is fast and simple, however, insufficient sensitivity limits its usage in clinical settings. Nowadays, molecular techniques are becoming widely used for identification of respiratory etiologies in clinical practice. Multiplex PCR can detect several targets at one time, however, design and optimization of dozens of primers and probes remain challenging. Microarray has the benefits of high-throughput, speed and low-cost. Suspension microarray enables simultaneously detect a number of pathogens in a single assay, it has faster hybridization kinetics and more flexibility in array construction compared to traditional solid-phase array.

A variety of sampling methods have been used in detecting respiratory pathogens in clinical practice. Recently, several studies have described the performance of nasal swab, nasal wash, nasopharyngeal aspirate, nasopharyngeal swab (NPS), oropharyngeal swab (OPS), sputum and bronchoalveolar lavage fluid (BALF) samples in microbiological findings using qRT-PCR or multipathogen detection platforms [5–9]. When testing for respiratory agents, the recommended and commonly collected sample is an upper respiratory swab because nasopharynx and oropharynx are two of the most common portals for the introduction of microbes into the respiratory tract. However, collection of NPS is an uncomfortable sampling method for young children, particularly pediatric patients with nasal congestion. Compared to NPS, OPS is less technically challenging and more acceptable to children because it is quick and simple [10]. In addition, some reports have showed that OPS can increase the number of viral infections identified by 15%, compared to the NPS alone [11]. Specifically, it has been found to be significantly more sensitive than NPS for the detection of certain viruses, such as adenovirus and 2009 pandemic influenza A (H1N1) virus [12]. So far, few studies have compared BALF and paired OPS samples from young children hospitalized with RTI.

In the present study, we used the suspension microarray, a multipathogen detection platform, to simultaneously detect viral and bacterial respiratory pathogens in matched OPS and BALF specimens from pediatric patients for comparison of the sensitivities between the two sample types.

## Methods

### Patients and specimen collection

The study protocol was approved by the Institutional Ethics Board of Dongguan Maternal and Child Health Care Hospital, China. Written informed consent for the BALF procedure and OPS sampling was obtained from each parent or guardian. Children aged 1 month to 7 years with signs and symptoms of RTI admitted to the study hospital between October 2017 and September 2018 were enrolled into this study. OPS and BALF specimens were collected within 3–5 days of hospital admission. First, we collected the OPS samples, a cotton-tipped swab was used to swab over the posterior pharynx and tonsils. BALF specimens were obtained with bronchoscope according to the 2009 and 2018 guidelines of pediatric bronchoscopy in China. Samples were stored at 4 °C until analysis and all specimens were tested within 24 h of collection.

### Nucleic acid extraction

Nucleic acid was isolated from BALF and OPS specimens using a magnetic bead-based Blood Total Nucleic Acid Kit (MagCares™, #M1701, Genehar Technologies Inc., Guangzhou, China) according to the manufacturer’s protocol. DNA and RNA concentrations were measured using a Quawell Q5000 UV spectrophotometer.

### Suspension microarray analysis

To detect multiple respiratory pathogens in a single assay, suspension microarray was developed in-house based on the Luminex xMAP system. The procedure consisted of multiplex PCR, probe design, the attachment of probes to microspheres and hybridization as previously reported [13, 14]. The targets (8 viruses without subtyping and 5 bacteria) included in suspension microarray are: influenza A (INF-A), influenza B (INF-B), parainfluenza viruses (PIV), human metapneumovirus (HMPV), human rhinovirus (HRV), respiratory syncytial virus (RSV), human bocavirus (HBov), adenovirus (Adv), *Streptococcus pneumoniae* (SP), *Mycoplasma pneumoniae* (MP), *Moraxella catarrhalis* (MC), *Haemophilus influenzae* (Hi) and *Bordetella pertussis* (BP).

### Statistical analysis

To assess the sensitivity for each sampling method, the presence of a pathogen in either of the specimens was deemed to be a true positive [15, 16]. We compared the differences in sensitivity for each pathogen between two sample types using the Chi-squared test or Fisher’s exact test, as appropriate. Statistical analyses were conducted with the software GraphPad Prism 5.0. A two-tailed *p*-value < 0.05 was considered statistically significant.

## Results

### Clinical information of included subjects

A total of 76 young children were subjected to respiratory pathogens detection in this project. Clinical characteristics of the patients are summarized in Table 1. The patients included 50 males and 26 females. The median age of children was 16 months (range 1 month to 7 years), and 84.2% of included children were younger than 5 years old. According to the 2013 WHO definition of severe pneumonia and guideline for children with community acquired pneumonia in China (2013 version), 22 patients were considered as severe respiratory tract infection (SRTI) and admitted to PICU with clinical presentation of cough, difficulty in breathing/tachypnoea, and one or more of the general danger signs such as an inability to drink, persistent vomiting, convulsions, lethargy or unconsciousness, central cyanosis.

**Table 1:** Demographic and clinical characteristics of 76 cases

Category	Subcategory	Number (%)
Gender	Male	50 (65.8%)
	Female	26 (34.2%)
Age	Range	1 month −7 years
	Median	16 months
	< 1 year	30 (39.5%)
	1–5 years	34 (44.7%)
	5–7 years	12 (15.8%)
Clinical presentation	Cough	76 (100%)
	Fever	51 (67.1%)
	Wheeze	36 (47.3%)
	Tachypnoea	4 (5.3%)
	Cyanosis	3 (3.9%)
	Hemoptysis	2 (2.6%)
Laboratory testing	White blood cell	76 (100%)
	C-reaction protein	72 (94.7%)
	Procalcitonin	70 (92.1%)
	Sputum culture	42 (55.3%)
Radiography	Chest X-ray	15 (19.7%)
	Computed tomography	61 (80.3%)
Diagnosis	Bronchopneumonia	42 (55.3%)
	Severe pneumonia admitted to PICU	22 (28.9%)
	Bronchiolitis	8 (10.5%)
	Chronic cough	3 (3.9%)
	Obliterative bronchiolitis	1 (1.3%)
Management	Supplemental oxygen	47 (61.8%)
	Mechanical ventilation	9 (11.8%)

*PICU* pediatric intensive care unit

### Pathogens identified in OPS and paired BALF

To compare the OPS and paired BALF for pathogens detection in young children with RTI, we tested the two sample types using suspension microarray. Of the 76 cases included in the analysis, 62 patients (81.6%) had at least one respiratory pathogen. BALF and OPS identified respiratory pathogen infections in 57 (75%) and 49 (64.5%) cases, respectively (Table 2). There was not a significant difference in detection rates between the two sample types (*p* > 0.05). Of these, 39 (51.3%) had concordant OPS and BALF suspension array results: 25 (32.9%) had the same pathogens identified from the OPS and BALF suspension array, and 14 (18.4%) had concordant negative results.

**Table 2:** Comparison of detection rates between two sample types

	No. (%) of bronchoalveolar lavage fluid	
	Positive	Negative
No. (%) of oropharyngeal swab		
Positive	44 (57.9%)	5 (6.6%)
Negative	13 (17.1%)	14 (18.4%)

The overall distribution of the respiratory pathogens tested is shown in Table 3. A total of 97 pathogens were found in 76 young patients. The etiology analysis revealed that viruses were responsible for 53.7% of patients, whereas bacteria accounted for 32.9% and *Mycoplasma pneumoniae* for 13.4%. The leading 5 pathogens identified were RSV, SP, Hi, MP and Adv, and they accounted for 74.2% of etiological fraction. For detection of any pathogen, the overall detection rate of BALF (81%) was marginally higher than that (69%) of OPS (*p* = 0.046). Also, BALF was more sensitive than OPS for detecting *Moraxella catarrhalis* (*p* < 0.05). The differences in frequency distribution and sensitivity for each single pathogen except *Moraxella catarrhalis* of two sampling methods were not statistically significant.

**Table 3:** Etiological distribution and sensitivity for all pathogens detected by two methods

Pathogens	Total	Oropharyngeal swab		Bronchoalveolar lavage fluid		*P* value^a^
		No. identified	Sensitivity (95% CI)	No. identified	Sensitivity (95% CI)	
RSV	19	15	79 (54–94)	16	84 (60–97)	NS
SP	17	14	82 (57–96)	10	59 (33–82)	NS
Hi	16	12	75 (48–93)	11	69 (41–89)	NS
MP	13	8	62 (32–86)	12	92 (64–100)	NS
Adv	7	4	57 (18–90)	7	100 (59–100)	NS
BP	5	3	60 (15–95)	4	80 (28–99)	NS
HBov	5	3	60 (15–95)	4	80 (28–99)	NS
PIV	4	4	100 (40–100)	3	75 (19–99)	NS
MC	4	0	0 (0–60)	4	100 (40–100)	< 0.05
INF-A	3	1	33 (1–91)	3	100 (29–100)	NS
INF-B	2	1	50 (1–99)	1	50 (1–99)	NS
HMPV	2	1	50 (1–99)	2	100 (16–100)	NS
All pathogens	97	67	69 (59–78)	79	81 (72–88)	< 0.05

*NS* non-significant

*Adv* adenovirus, *HBoV* human bocavirus, *HMPV* human metapneumovirus, *INF-A* influenza A, *INF-B* influenza B, *PIV* parainfluenza viruses, *RSV* respiratory syncytial virus, *BP Bordetella pertussis*, *Hi Haemophilus influenzae*, *MC Moraxella catarrhalis*, *MP Mycoplasma pneumoniae*, *SP Streptococcus pneumoniae*

^a^ Chi-squared test or Fisher’s exact test

### Distribution of pathogens in patients with co-infections

Virus/virus and virus/bacterium co-infections were found in OPS and BALF (Table 4). In OPS, a single pathogen was found in 34 cases (69.3%), two pathogens in 13 cases (26.6%), and three pathogens in 2 cases (4.1%). In BALF, a single pathogen was identified in 41 cases (71.9%), two pathogens in 13 cases (22.9%), three pathogens in 2 cases (3.5%), and four agents in 1 case (1.7%). The most common co-infections observed was SP plus MP (*n* = 3) in OPS samples, RSV plus Hi (n = 3) and followed by RSV plus SP (*n* = 2) in BALF specimens.

**Table 4:** Distribution of pathogens in patients with co-infections

Oropharyngeal swab (No.)	Bronchoalveolar lavage fluid (No.)
SP + MP (3)	RSV + Hi (3)
Hi + PIV (1)	RSV + SP (2)
Hi + Adv (1)	SP + MP (1)
BP + RSV (1)	BP + RSV (1)
RSV + PIV (1)	MP + RSV (1)
HBoV+SP (1)	RSV + Adv (1)
HBoV+RSV (1)	HMVP+SP (1)
SP + INF-B (1)	Hi + MC (1)
Hi + SP (1)	PIV + MP (1)
Hi + RSV (1)	SP + MC (1)
SP + HMPV (1)	RSV + PIV + MC (1)
Hi + BP + RSV (1)	RSV + HBoV+SP (1)
Hi + SP + INF-A (1)	Hi + Adv + MP + MC (1)
Total (15)	Total (16)

*Adv* adenovirus, *HBoV* human bocavirus, *HMPV* human metapneumovirus, *INF-A* influenza A, *INF-B* influenza B, *PIV* parainfluenza viruses, *RSV* respiratory syncytial virus, *BP Bordetella pertussis*, *Hi Haemophilus influenzae*, *MC Moraxella catarrhalis*, *MP Mycoplasma pneumoniae*; SP: *Streptococcus pneumoniae*

## Discussion

In the present study, we used suspension array to simultaneously detect multiple viral and bacterial pathogens in paired BALF and OPS specimens from symptomatic patients hospitalized with respiratory illness. To validate the reliability and accuracy of the multipathogen testing platform, we have compared the yield of suspension array with that of metagenomic next-generation sequencing for microbiological findings and highlighted the high concordance of 13 targets between the two methods (manuscript in preparation). These results showed that our suspension array can reliably identify pathogens in patients with RTI. The fast turnaround time (within 4–5 h) makes it possible to be a valuable tool in clinical settings.

The reliability of the specimens taken via oropharyngeal/nasopharyngeal swab or wash compared to the deep samples such as BALF is a matter of debate and it would be interesting to investigate if they are really useful. Here, BALF and OPS had similar microbiological yields (75% vs. 65%, *p* > 0.05). The differences in the frequency distribution and sensitivity for most targeted pathogens except *Moraxella catarrhalis* of two sampling methods were not statistically significant. Selection of a sampling method for detecting respiratory pathogens must take into account its sensitivity, feasibility and costs. The collection of OPS is relatively simple, quick and less invasive compared to other sampling methods. For these reasons, we consider the OPS as the preferred method of respiratory tract sampling for pathogen detection.

For *Moraxella catarrhalis*, we detected 4 cases in BALF and none in paired OPS specimens. In general, *Moraxella catarrhalis* causes mainly upper respiratory tract infection (otitis media) in children and lower respiratory tract infection in adults with previously compromised airways such as chronic obstructive pulmonary disease [17, 18]. However, some reports have demonstrated that *Moraxella catarrhalis* may be involved in lower respiratory tract infections in children [19], which is consistent with our results. More studies are needed to investigate its role in respiratory illness in hospitalized children.

Among the 76 patients, 25 (32.9%) had the same pathogens identified from the OPS and BALF suspension array, and 14 (18.4%) had concordant negative results. For the 14 cases, they might be infected by some rare pathogens that are not covered by our suspension array. The top 5 pathogens were RSV, SP, Hi, MP and Adv, accounting for 74.2% of etiological fraction. Our data are in agreement with other recent findings in multi-country case-control studies that found RSV was the most common cause of severe childhood pneumonia [20, 21]. Thus, RSV could be a primary target for children hospitalized with respiratory illness. In general, RSV is most commonly found in lower respiratory tract infections particularly in infants [22]. However, the sensitivities of RSV between OPS and BALF was not significantly different in our testing. RSV infection and replication initiates in the nasopharynx. The virus could be found in both upper and lower airway via high-sensitivity molecular techniques when it spreads from the upper respiratory tract to the lower in individuals with compromised immunity.

*Bordetella pertussis* was rarely identified in infants perhaps due to high vaccination rates. In this study, we detected *Bordetella pertussis* in 5 young children. Identification of *Bordetella pertussis* in BALF or OPS specimens may provide predictive value for the outcome of respiratory illness at the individual case level [23]. In terms of some pathogens such as influenza A, adenovirus, *Mycoplasma pneumoniae* and *Streptococcus pneumoniae*, relatively low concordance between BALF and OPS specimens for them may reflect different cell tropisms for different parts of the respiratory tract. Notably, none of the 76 cases was tested positive for HRV in this work, which is somewhat surprising given that this virus is often associated with upper respiratory infection. However, another project in our group showed that the detection rate of HRV was ~ 2% in OPS among 2895 pediatric outpatients with respiratory illness. Here, the included children were inpatients. Although HRV infections are frequent, they are mostly limited to the upper respiratory tract and generally cause relatively mild symptoms [24, 25]. The contribution of HRV may vary by disease severity of included patients and other factors.

Cultivation is regarded as the gold standard in etiological identification. As shown in Table 1, we have performed sputum culture in 42 of 76 cases. Compared with PCR-based methods, the detection rate of it was significantly lower. One of possible explanations is the empirical antibiotic therapy in patients before sampling. Here, we focused on comparison of OPS and paired BALF in detecting respiratory pathogens, rather than the sensitivity of nucleic acid-based array compared to the gold standard, i.e. cultivation. Based on the same consideration, a healthy control group was not tested for ruling out false positives in this work because of our specific interest and aim. Similarly, a prior study, 86 patients enrolled and no healthy controls included, has also applied this strategy to compare the yields of bronchoalveolar lavage samples with that of nasopharyngeal swabs by using FilmArray respiratory panel [8].

This study has several limitations. First, our sample size was relatively small (*n* = 76) because we focused on paired BALF and OPS specimens collected from hospitalized children. As a result, there was limited power to compare the sensitivities of BALF and OPS for specific respiratory pathogens in patients. Further studies in a larger cohort may generate a relatively high degree of precision when performing comparative statistical analysis. Second, although BALF are regarded lower respiratory tract samples, oropharyngeal intubation for BALF might result in potential contamination by upper respiratory tract “contaminants”, particularly for bacteria/viruses known to colonize the oropharynx. Thus, the BALF specimen might be actually both an upper and a lower airway combined sample. However, it does not affect our primary purpose that focuses on the microbiological findings of OPS sampling. Third, empirical antibiotic use in clinical practice may reduce the sensitivity of assays, particularly for bacteria. Collectively, identification of a pathogen does not necessarily equate to the etiological agent, particularly in a multipathogen testing and laboratory results require further interpretation by experienced clinicians. In addition, we here focused on a subset of potential pathogens because the 13 agents (8 viruses and 5 bacteria) are key respiratory pathogens in children based on previous epidemiological investigations in China. In fact, fungi are also important pathogens causing severe infections of the respiratory system. Another project in our group by using metagenomic next-generation sequencing found that the infection rates of *Candida albicans*, *Pneumocystis jiroveci* and *Aspergillus fumigatus* in young children admitted to PICU with respiratory illness were 3.52, 1.68 and 1.23%, respectively. Given the high prevalence and importance of the airborne fungal pathogens in respiratory infections, we plan to add fungal species to our upgraded in-house array, which would be a separate study due to many experiments and large undertakings.

## Conclusions

We used suspension-array to compare BALF and paired OPS specimens for detecting multiple pathogens in children hospitalized with respiratory illness. The similar sensitivities between the two sampling methods indicated the clinical value of OPS testing in clinical settings.

## Data Availability

The data set used and/or analyzed during the current study is available from the corresponding author on reasonable request.

## Abbreviations

Adv: Adenovirus
BALF: Bronchoalveolar lavage fluid
BP: *Bordetella pertussis*
HBov: Human bocavirus
Hi: *Haemophilus influenzae*
HMPV: Human metapneumovirus
HRV: Human rhinovirus
INF-A: Influenza A
INF-B: Influenza B
MC: *Moraxella catarrhalis*
MP: *Mycoplasma pneumoniae*
NPS: Nasopharyngeal swab
OPS: Oropharyngeal swab
PICU: Pediatric intensive care unit
PIV: Parainfluenza viruses
RSV: Respiratory syncytial virus
RTI: Respiratory tract infection
SP: *Streptococcus pneumoniae*
SRTI: Severe respiratory tract infection
